# The complete mitochondrial genome of the file ramshorn snail *Planorbella pilsbryi* (Mollusca: Gastropoda: Hygrophila: Planorbidae)

**DOI:** 10.1080/23802359.2021.1975508

**Published:** 2021-10-11

**Authors:** Emma M. Rempel, Jeffrey M. Marcus, Jillian T. Detwiler

**Affiliations:** Department of Biological Sciences, University of Manitoba, Winnipeg, MB, Canada

**Keywords:** Illumina sequencing, mitogenomics, Planorbidae, nuclear rRNA repeat, phylogeny

## Abstract

The file ramshorn snail *Planorbella pilsbryi* Baker, 1926 (Gastropoda: Hygrophila: Planorbidae) is a widespread herbivorous North American freshwater snail found in diverse habitats, including standing and moving water bodies. Genome skimming by Illumina sequencing allowed the assembly of a complete nuclear rRNA repeat sequence and a complete circular mitogenome of 13,720 bp from *P. pilsbryi* consisting of 75.3% AT nucleotides, 22 tRNAs, 13 protein-coding genes, 2 rRNAs and a control region in the typical order found in panpulmonate snails. *Planorbella pilsbryi COXI* features a rare TTG start codon while *COXII, CYTB, ND2, ND3,* and *ND5* exhibit incomplete stop codons completed by the addition of 3′ A residues to the mRNA. Phylogenetic reconstruction of mitochondrial protein-coding gene and rRNA sequences places *P. pilsbryi* as sister taxon to *Planorbella duryi* (Planorbidae) within family Planorbidae, which is consistent with previous phylogenetic hypotheses.

The file ramshorn snail, *Planorbella pilsbryi* Baker 1926 is a common freshwater snail species native to southern Canada and the United States (Baker 1945; Clarke 1981; McKillop 1996). These snails play vital roles in a variety of environments as principal grazers, contributors to nutrient cycling, and prey for invertebrate and vertebrate predators (Baker 1945; Johnson et al. 2013). Furthermore, they are used in toxicology studies to determine the effects of herbicides and insecticides on freshwater invertebrate communities (Prosser et al. 2016). However, taxonomic questions have been raised that may have implications for interpretation of ecological studies. For example, some taxonomists suggest that *P. pilsbryi* is a genetic variant and eco-phenotype of *Planorbella trivolvis,* rather than a distinct species (Pip 1987). To better understand the taxonomy and evolutionary ecology of *P. pilsbryi*, we characterized its mitogenome and nuclear rRNA repeat.

Here we report the complete mitochondrial genome sequence of *P. pilsbryi* from specimen PlPi_MW889961, collected from the Marais River in Manitoba, Canada (GPS 49.127 N, 97.303 W) on 11 June 2019 that was deposited in the collection of the Manitoba Museum (https://manitobamuseum.ca/collections-research/, Randall Mooi, rmooi@manitobamuseum.ca), voucher MM 67397. The specimen was identified as *P. pilsbryi* using criteria outlined by Baker (1945), namely a wide, bowl-like spire, a deeply excavated umbilical region, and a shell height equal to roughly 50% of the shell’s diameter. DNA was extracted from 30 mg of tissue from the head-foot region using an E.Z.N.A Mollusk DNA kit **(**Omega Bio-tek Inc., Norcross, USA) before it was sheared by sonication and a fragment library was prepared using the NEBNext Ultra II DNA Library Prep Kit for Illumina (New England Biolabs, Ipswich, Massachusetts) as previously described (Peters and Marcus 2017). Finally, it was sequenced by Illumina NovaSeq6000 (San Diego, California, USA) at Genome Quebec.

Mitogenome assembly of *P. pilsbryi* (Genbank accession MW889961) was performed by mapping the resulting sequence library of 21,753,838 paired 150 bp reads (Genbank SRA PRJNA718624) to a *Planorbella duryi* reference mitogenome (Gastropoda: Planorbidae, KY514384 (Schultz et al. 2018)) using 5 iterations of the medium sensitivity settings of Geneious 2020.2 as described by Marcus (2018). Annotation was in reference to *P. duryi* and *Biomphalaria choanomphala* (Hygrophila: Planorbidae, MG431964) mitogenomes (Zhang et al. 2018). The complete *P. pilsbryi* nuclear rRNA repeat (8182 bp) (MW883067) was also assembled and annotated using reference sequences from the *18S rRNA* gene from *Galba cubensis* (Gastropoda: Lymnaeidae, Z83831) (Bargues et al. 1997); the partial *18S rRNA*, *ITS1*, *5.8S rRNA*, *ITS2*, and partial *28S rRNA* region from *Satsuma polymorpha* (Gastropoda: Camaenidae, AB597368) (Hoso et al. 2010); the *28S rRNA* genes from *P. trivolvis* (KY319366) (unpublished) and *Pomacea bridgesi* (Gastropoda: Ampullariidae, DQ279984) (Giribet et al. 2006); and the complete rRNA repeat from *Macrosoma conifera* (Lepidoptera: Hedylidae, MT878224) (McCullagh et al. 2020).

The *P. pilsbryi* circular 13,720 bp mitogenome assembly was composed of 5,620 paired reads with nucleotide composition: 33.8% A, 11.4% C, 13.3% G, and 41.5% T. The gene composition and order in *P. pilsbryi* is identical to all known panpulmonate snails except for *Physella acuta* (Physidae) (Nolan et al. 2014). Protein-coding gene start codons in the *P. pilsbryi* mitogenome include ATG (*COXIII*, *ND1*, *ND3*, and *ND5*), ATT (*ATP6*, *ATP8*, and *ND2*), ATA (*COXII*, *CYTB*, *ND4*, and *ND6*), ATC (*ND4L*), and TTG (*COXI*). The mitogenome contains 7 protein-coding genes (*ATP6*, *ATP8*, *COXI*, *COXIII*, *ND1*, *ND4*, and *ND6*) with TAA stop codons, 1 protein-coding gene (*ND4L*) with a TAG stop codon, and 5 protein-coding genes (*COXII*, *CYTB, ND2, ND3,* and *ND5*) with single-nucleotide (T) stop codons completed by post-transcriptional addition of 3′ A residues. The locations and structures of tRNAs were determined using ARWEN v.1.2 (Laslett and Canback 2008). All 22 tRNAs have typical cloverleaf secondary structures except for: trnG (TCC) where the dihydrouridine arm (D-arm) is replaced by a loop (D-replacement loop), as well as trnS1 (TGA), trnS2 (GCT), and trnLA (TTT), where the TφC (T) arm and variable (V) loop are replaced by a loop (TV-replacement loop). The nuclear rRNA sequences increase the number of genetic loci that are available for future use in phylogenetic analyses of the Planorbidae.

We reconstructed a phylogeny using 11 protein-coding genes and 2 rRNA genes from *P. pilsbryi*, 17 gastropod species within the superorder Hygrophila, and the gastropod *Pyramidella dolabrata* (superfamily Pyramidelloidea) as an outgroup (Grande et al. 2008). Two protein-coding genes (*ATP6* and *ATP8*) were excluded to increase taxon sampling in the phylogeny. Mitogenome sequences were aligned in CLUSTAL Omega (Sievers et al. 2011). The best fit model of evolution was GTR + I + G according to jModeltest 2.1.7 (Darriba et al. 2012). For maximum likelihood analysis, we employed the GTRGAMMA model and calculated bootstrap probabilities with 1,000 replicates in RaxML v8 (Stamatakis 2014). For Bayesian inferences, 2 independent analyses were run with 4 chains each for 10,000,000 generations with samples taken every 1,000 generations using MrBayes v3.2.6 (Ronquist and Huelsenbeck 2003). A burn-in of 2,500 was used and the remaining trees were used to calculate the Bayesian posterior probabilities. Phylogenetic analysis supports the monophyletic intrageneric relationship between *P. duryi* and *P. pilsbryi* (Figure 1). The mitogenome of *P. pilsbryi* can be used to clarify the evolutionary relationships among gastropod taxa at higher (family) and lower (species) levels of taxonomy. Further, this resource can help ground ecological investigations that include *Planorbella* species in a clearer taxonomic framework.

**Figure 1.**
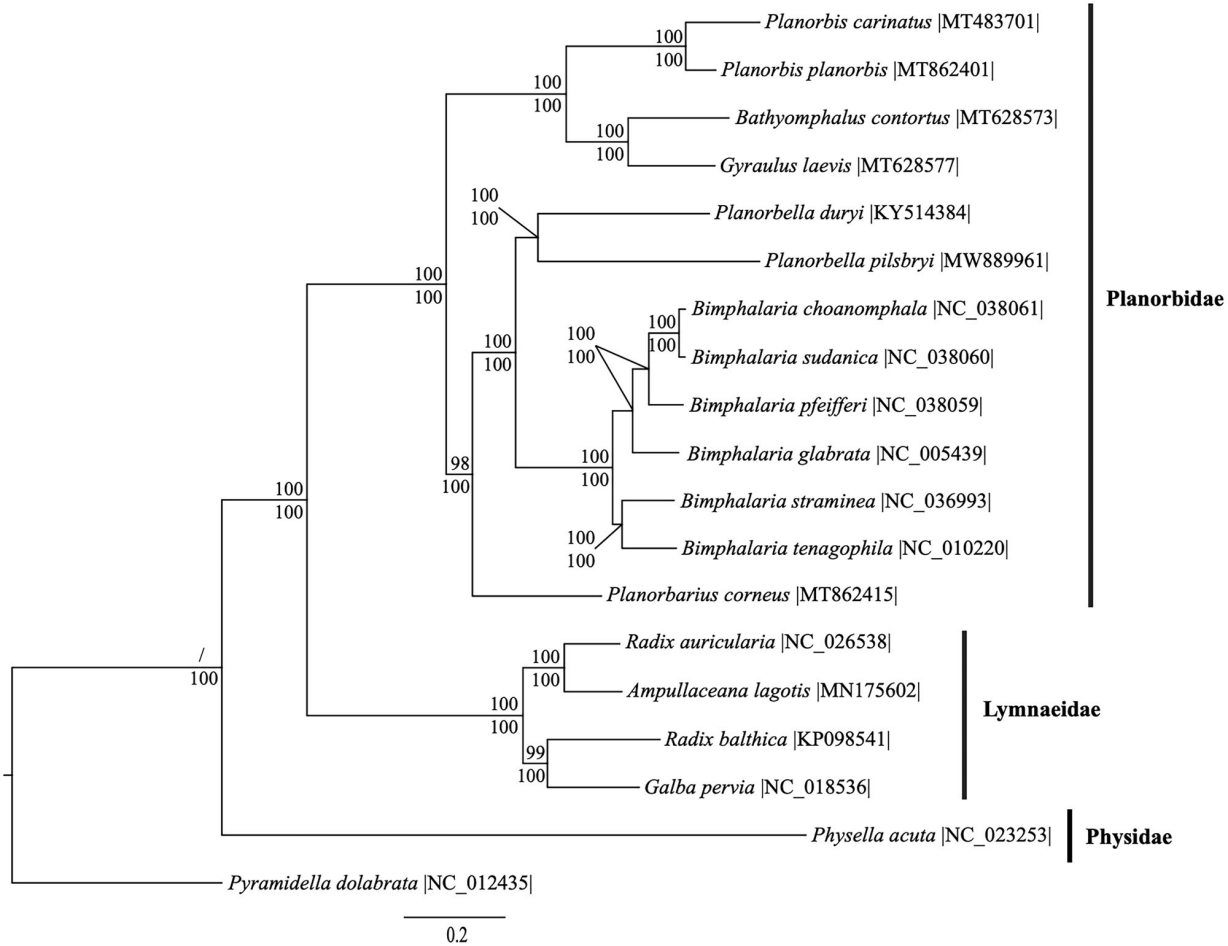
Phylogenetic reconstruction of mitogenomes (11 protein-coding genes and 2 rRNA genes) of *Planorbella pilsbryi*, 17 additional sequences from superorder Hygrophila, and 1 outgroup species, *Pyramidella dolbrata,* from superfamily Pyramidelloidea. Sequences were analyzed using maximum likelihood (ML) and Bayesian inference (BI). The Bayesian inference tree is shown. Numbers above each node are ML bootstrap values, while Bayesian posterior probabilities are displayed under each node.

## Data Availability

The data that support the findings of this study are openly available in GenBank of NCBI at [https://www.ncbi.nlm.nih.gov] (http://www.ncbi.nlm.nih.gov/) under the accession nos. MW889961 and MW883067. The associated BioProject, SRA, and Bio-Sample numbers are PRJNA718624, SRX10533846, and SAMN18543613. The specimen was deposited in the Manitoba Museum (https://manitobamuseum.ca/collections-research/, Randall Mooi, rmooi@manitobamuseum.ca), voucher MM 67397.
